# Solubility, Permeability, and Dissolution Rate of Naftidrofuryl Oxalate Based on BCS Criteria

**DOI:** 10.3390/pharmaceutics12121238

**Published:** 2020-12-19

**Authors:** Marta Kus-Slowinska, Monika Wrzaskowska, Izabela Ibragimow, Piotr Igor Czaklosz, Anna Olejnik, Hanna Piotrowska-Kempisty

**Affiliations:** 1 Department of Toxicology, Poznan University of Medical Sciences, 30 Dojazd St., 60-631 Poznan, Poland; marta.kus-slowinska@ethifarm.pl; 2 Research and Development Department of Ethifarm, Ethifarm Manufacturing Plant, 9 Stefana Zeromskiego St., 60-544 Poznan, Poland; monika.wrzaskowska@ethifarm.pl (M.W.); izabela.ibragimow@ethifarm.pl (I.I.); piotr.czaklosz@ethifarm.pl (P.I.C.); 3 Department of Biotechnology and Food Microbiology, Poznan University of Life Sciences, 48 Wojska Polskiego St., 60-627 Poznan, Poland; 4 Department of Basic and Preclinical Sciences, Institute of Veterinary Medicine, Nicolaus Copernicus University in Toruń, 7 Gagarina St., 87-100 Torun, Poland

**Keywords:** naftidrofuryl oxalate, solubility, permeability, dissolution profiles, pharmaceutical availability, BCS drug classification

## Abstract

The Biopharmaceutics Classification System (BCS) was conceived to classify drug substances by their in vitro aqueous solubility and permeability properties. The essential activity of naftidrofuryl oxalate (NF) has been described as the inhibition of the serotonin receptors (5-HT_2_), resulting in vasodilation and decreasing blood pressure. Since the early 1980s, NF has been used to treat several venous and cerebral diseases. There is no data available on the BCS classification of NF. However, based on its physical-chemical properties, NF might be considered to belong to the 1st or the 3rd BCS class. The present study aimed to provide data concerning the solubility and permeability of NF through Caco-2 monolayers and propose its preliminary classification into BCS. We showed that NF is a highly soluble and permeable drug substance; thus, it might be suggested to belong to BCS class I. Additionally, a high dissolution rate of the encapsulated NF based on Praxilene^®^ 100 mg formulation was revealed. Hence, it might be considered as an immediate-release (IR).

## 1. Introduction

Pharmacokinetics characterizes the drug’s behavior in the body from its administration to excretion. It describes five major physical and chemical properties: liberation, absorption, distribution, metabolism, and elimination (LADME). The first two stages of LADME are characterized by the Biopharmaceutical Classification System (BCS), introduced by Amidon et al. in 1995 [1]. BCS is a scientific framework for classifying drug substances based on their aqueous solubility and intestinal permeability, which largely allows the prediction of a given drug substance bioavailability (BA) in human blood plasma. The BA of drugs depends mainly on their physicochemical properties (solubility, dissociation, and lipophilicity), dosage form (influence of excipients on active pharmaceutical ingredients (API) properties), and the route of administration (oral—liver metabolism) [2,3].

Naftidrofuryl hydrogen oxalate (NF, C_24_H_33_NO_3_, C_2_H_2_O_4_; synonym: nafronyl oxalate), as a pharmacologically active drug compound, is a mixture of four stereoisomers [4]. NF is a white powder freely soluble in water and ethanol (with pKa 8.2 at 30 °C) [5,6]. It selectively inhibits 5-hydroxytryptamine type 2A (serotonin; type 5-HT_2A_) receptors present in vascular smooth muscle cells, platelets, and endothelial cells (in vascular and cerebral tissues). In vascular cells, NF causes vascular vasodilation (by preventing smooth muscle contraction) and decreases blood pressure [7]. Furthermore, it inhibits serotonin-induced platelet aggregation and platelet- induced vasospasm [8]. NF has also been shown to increase the ATP level in fibroblast and endothelial cells by improving the aerobic metabolism in the blood vessel wall [4]. As a drug product, NF has been mainly used for the treatment of intermittent claudication (since 80th) [9], less often also for stroke [10] and dementia [11].

A literature review of NF pharmacokinetics properties is detailed in Table 1. The main NF capsules manufacturer states that the peak plasma concentration (C_max_) occurs about 30 min after oral dosing [12]. The relative BA of 100 mg NF administered orally was estimated 121% (oral solution containing 100 mg of NF as a reference was used) [6]. Furthermore, based on the pharmacokinetic data presented by Brodie et al., the high relative BA of NF (approx 100% following oral administration of 100 mg tablets) can be suggested [13]. However, the results of several studies about BA of NF are controversial. Nishigaki et al. showed that the relative bioavailability of 100 mg NF after its oral administration was within the 20–23% range. The authors concluded that this was due to the large first-pass elimination metabolism (80%) [14]. Additionally, the pharmacokinetic analysis performed in the dogs has also demonstrated a low absolute BA of 250 mg NF in the range of 0.3–2.7% due to presystemic and/or first-pass metabolism [15].

After absorption, NF is distributed and stored predominantly in fatty tissue [16]. NF is primarily biotransformed in hepatocytes to three acidic metabolites, among which NF free acid (3-(1-naphthyl)-2 tetrahydrofurfuryl propionic acid) is the main one [17,18]. Less than 1% of absorbed NF is excreted in urine within 48 h of administration, while most of it appears to be eliminated with the bile [19].

**Table 1:** Summary of literature data on the NF pharmacokinetics.

Dosage Form	Dosage [mg]	No. of Patients	T_1/2_ [h]	T_max_ [h]	C_max_ [ng/mL]	AUC [ng/mL*h]	BA [%]	Reference
**oral**	nd	nd	nd	nd	nd	nd	30	[20]
**oral (gelatin capsules)**	50	n/d	0.68	1.1	350	nd	76	[21]
**oral (capsules)**	100	2	1.2	0.5	203	nd	nd	[13]
**intravenous**	40	18	nd	nd	nd	nd	nd	[16]
**oral (gelatin capsules)**	300	12	1.79	0.94	922	2022	nd	[21]
**oral (fasted)** **oral (nonfasted)**	100	12	1.3 1.6	0.8 2	238 181	500 583	19.7 23.0	[14]
**oral (tablets)**	100 300 300	9	nd	1 0.9 1.2	209 590 645	630 1271 1955	121 77 114	[6]
**oral (tablets)**	200	30	3.41	2.75	279	1797	nd	[22]
**oral (tablets)**	200	12	4.4	1.3	174	1504	nd	[23]

nd = no data; T_1/2_ = half life; T_max_ = the time taken to reach the maximum concentration; C_max_ = the peak plasma concentration; AUC = area under curve; BA = bioavailability.

NF drug products are widely distributed all over the world. The clinical dose of naftidrofuryl is 100 or 200 mg for all its dosage forms (capsules and tablets).

To the best of the authors’ knowledge, there is no data concerning the permeability of NF or its BCS classification. Hence, to fulfill the literature gap, the present study aimed to provide data concerning aqueous solubility and permeability of NF through Caco-2 monolayers and propose its preliminary classification into BCS. We performed three experiments concerning the solubility, permeability, and dissolution of NF largely based on the BSC guidelines for the pharmaceutical industry [24,25]. However, industry guidelines are mainly focused and directed for biowaiver approach applicants. Hence, in the present study, several issues in experimental design and data evaluation differ from the formal requirements.

## 2. Materials and Methods

### 2.1. Reagents and References

The acetonitrile (ACN; VWR Chemicals, Fontenay-sous-Bois, France) and water (H_2_O; VWR Chemicals, Fontenay-sous-Bois, France) for the HPLC assay were of chromatographic grade. Other reagents were of analytical grade: hydrochloric acid (HCl, Chempur, Piekary Slaskie Poland), disodium hydrogen phosphate (Na_2_HPO_4_; Chempur, Piekary Slaskie, Poland), ammonium acetate (CH_3_CO_2_NH_4_; Chempur, Piekary Slaskie, Poland), sodium acetate (CH_3_COONa; Chempur, Piekary Slaskie, Poland), glacial acetic acid (CH_3_COOH; Chempur, Piekary Slaskie, Poland), and orthophosphoric acid (85%; H_3_PO_4_; Chempur, Piekary Slaskie, Poland). The media solutions used for solubility and dissolution tests were prepared according to European Pharmacopoeia (EP) Chapter 4–Reagents.

A British Pharmacopoeia Certificated Reference Standard of Naftidrofuryl oxalate (BPCRS, Cat no. 362) was used for all the performed assays. Additionally, we used the Caffeine standard (CAF) (Sigma Aldrich–Merck Group, Darmstadt, Germany) for permeability tests as a reference. Praxilene^®^ 100 mg Capsules (Merck Serono Ltd., Feltham, UK) was employed as a drug reference product. Each Praxilene capsule contained 100 mg of naftidrofuryl oxalate.

Naftidrofuryl oxalate (Sigma Aldrich–Merck Group, Darmstadt, Germany), magnesium stearate (Peter Greven Nederland C.V., Venlo, Netherland), and talc (C.H. Erbslöh, Krefeld, Germany) used for the in-house capsules were obtained from Ethifarm (Ethifarm, Poznan, Poland). Hard gelatin capsules, size 3 (Capsugel, Morristown, New Jersey, NY, USA) used in the drug dosage manufacture were provided by Ethifarm (Ethifarm, Poznan, Poland).

### 2.2. Solubility Test

The solubility by equilibrium method was performed in three different media (0.1 M HCl, acetate buffer, and phosphate buffer), with a pH of 1.2, 4.5, and 6.8, respectively, based on European Medicines Agency (EMA) guideline [24]. Saturated solutions of NF in given media were prepared by adding an excessive amount of the drug until obtained a saturated solution (presence of visible precipitate on the tube). The samples were then incubated at 37 °C ± 0.5 (incubator chamber; Binder GmbH, Tuttlingen, Germany) for 24 h in an orbital agitation of 45 rpm (LLG-uniLOOPMIX 2 orbital shaker, LLG GmbH, Hamburg, Germany). After equilibration, the sample was filtered (0.45 µm) and diluted with the appropriate solution to the concentration within the linear calibration range. Drug concentration measurement was performed by a validated UV spectrophotometric method at λ = 283 nm with optical path 0.2 cm and spectrum recorded on 250–350 nm range (JASCO V-530 double-beam apparatus with Spectra Manager software, JASCO, Tokyo, Japan). The experiments were carried out in triplicate.

The dose number (D_0_) for each pH buffer solution was calculated using the following equation:

D_0_ = DOSE/V_0_/S (1)

where DOSE is the highest dose strength (mg), V_0_ is the water volume (assumed to be 250 mL), and S is the aqueous solubility (mg/mL). Drug substances with D_0_ ≤ 1 are classified as high solubility drugs, while drugs with D_0_ ≥ 1 are assigned as low solubility drugs [26].

### 2.3. Cytotoxicity Analysis

The MTT (3-(4,5-dimethyl-2-thiazolyl)-2,5-diphenyl-2H-tetrazolium bromide) test (Sigma Aldrich–Merck Group, Darmstadt, Germany) was used to establish an appropriate concentration of analyzed substances NF and CAF in the Caco-2 cell line. To determine the effects of materials tested on cell viability, confluent stock cultures were detached using trypsin and seeded in 96-well plates at a density of 2 × 10^4^ cells/well. They were allowed to attach overnight and then exposed for 2 h (time of transport across monolayer) for seven different NF/CAF concentrations in Hank’s Balanced Salt Solution (HBSS, Sigma Aldrich–Merck Group, Darmstadt, Germany) pH 7.4 in the range of 0.0 mg/mL to 1.0 mg/mL. After NF/CAF discarding, the mixture of growth medium and MTT (5 mg/mL) was added. Cells were incubated for 3 h until intracellular purple formazan crystals were visible under a microscope, and then dimethyl sulfoxide (DMSO), (POCH-Avantor, Gliwice, Poland) was added to each well to dissolve purple crystals of formazan. The absorbance was measured using a spectrophotometric method (BioTek U.S., Winooski, VT, USA) at a wavelength of 540 nm.

### 2.4. The Culture of Caco-2 Colon Cancer Cells

The colon cancer Caco-2 cell line (ATCC^®^ HTB-37™) was purchased from ATCC (American Type Culture Collection, Manassas, VA, USA) and used as the model of the epithelial intestinal permeability layer. Caco-2 cells were cultured in Dulbecco’s Modified Eagle’s Medium (DMEM, Merck Group, Darmstadt, Germany) supplemented with 1% nonessential amino acids mixture (MEM NON, Merck Group, Darmstadt, Germany), 20% fetal bovine serum (FBS, Merck Group, Darmstadt, Germany), and 2 mM L-glutamine, 100 U/mL penicillin and 0.1 mg/mL streptomycin solution (Merck Group, Darmstadt, Germany). For the transport monolayer preparation, the Caco-2 cells were seeded at 4 × 10^5^/cm^2^ into transparent membranes inserts with 0.4 µm pore size (Millipore–Merck Group, Darmstadt, Germany) in 6-wells plates (Corning-Life Science, Durham, NC, USA). The cells were cultured for 22 days at 37 °C in a humidified atmosphere containing 5% CO_2_ and 95% air. The integrity of the Caco-2 monolayer was evaluated by measuring the Trans Epithelial Electrical Resistance (TEER) every 48 h. The cells were considered to be suitable for use in the permeability study if the average TEER value was >600 Ohm × cm^2^ (Millicell ERS-2 Voltohmmeter, Millipore–Merck Group, Darmstadt, Germany).

### 2.5. Permeability Test

The permeability tests of NF and CAF solutions were performed in two directions: apical-to-basolateral (A–B) and basolateral-to-apical (B–A) in triplicate at 37 °C with shaking at 100 rpm for 2 h. For transport assay, drug substances (NF, CAF) were dissolved in the transport buffer solution (HBSS with 25 mM HEPES, pH 7.4) and applied in the apical or basolateral compartment in compliance with transport direction. At 15 min intervals, the sample from the acceptor compartment was taken during a 2-h transport experiment. Each sample volume (150 µL) was replaced with a fresh pre-heated (37 °C) transport buffer. The total solution volume in the apical and basolateral compartments was maintained at 2 mL and 4 mL, respectively. HPLC analysis was used to determine the concentration of the compound transported. Quantitative chromatographic analyses were performed using the reverse phase HPLC method and UV-VIS detector (Waters UPLC Acquity H-class apparatus with Empower 3.0 software, Waters Corporation, Milford, MA, USA). The analytical method of NF was based on British Pharmacopoeia monograph Naftydrofuryl capsules (with slight modifications) [27], while CAF was based on the European Pharmacopoeia monograph [5]. The chromatographic parameters for NF were set as follows: column (4.6 mm × 250 mm; 5 µm) filled with silica modified by phenyl groups (Spherisorb Phenyl columns, Waters Corporation, Milford, MA, USA). The liquid phase was a mixture of 55 volumes of ACN with 45 volumes of 0.05 M sodium acetate (pH 4.0 with 85% *v/v* orthophosphoric acid). The isocratic flow rate was set as 1 mL/min and the detection wavelength on λ = 283 nm. The retention time of Naftidrofuryl peak was about 10 min. Following the modifications, this chromatographic method was validated to be suitable for the assay. Transport data, including apparent permeability coefficient (P_app_) that reflects the rate at which compounds pass the intestinal barrier, were calculated according to the protocols described by Tavelin et al. [28] and Hubatsch et al. [29].

### 2.6. In-House NF Capsules Preparation

In-house 100 mg capsules with Naftidrofuryl oxalate were prepared as a test product (laboratory scale) for the NF dissolution profile assay. The in-house NF capsules were made according to the Praxilene^®^ 100 mg Capsules formulation, as presented in the Praxilene SPMC [12] in the proportions shown in Table 2.

### 2.7. In Vitro Dissolution Test and Data Evaluation

The release rate of NF (from in-house NF capsules vs. Praxilene^®^ 100 mg Capsules) was determined by using automated dissolution apparatus (European Pharmacopoeia, Type II; Erweka DT 828/1000 LH; Erweka GmbH, Langen, Germany) with paddles operating at a rotation speed of 50 rpm in 37 °C (±0.5 °C). The parameters for the dissolution test: 900 mL of three different dissolution media (0.1 M HCl, acetate buffer, phosphate buffer) with pH of 1.2, 4.5, and 6.8 respectively were used. Twelve in-house NF capsules (and 12 Praxilene units) were tested in each dissolution medium. The concentration values of the released NF were recorded at the following time points: 5, 10, 15, 20, 30, and 45 min. Samples (10 mL) of the tested solutions were withdrawn, then filtered (0.2 µm nylon syringe filter), and subsequently subjected to quantitative assessment by validated spectrophotometric method (as described in Section 2.2—Solubility test). The dissolution data were statistically evaluated using the two factor (f_1_, f_2_) method, according to the United States Food and Drug Administration (FDA) guideline [25]. As the first step, the spectrophotometric results were qualitatively evaluated (% RSD—relative standard deviation percentage) to ensure that they are suitable for the two factors calculation stage and, subsequently, for the dissolution profiles comparison and evaluation. The aggregated % RSD was calculated based on all the three dissolution media timepoints results obtained. However, the % RSD not exceeding 20% of the results obtained in the early time points (up to 10 min) and 10% for the subsequent timepoints is mandatory in this approach. The difference factor (f_1_) and similarity factor (f_2_) were calculated according to the FDA guidelines [25,30]. The two curves are considered to be similar if the value for f_1_ is not greater than 15% (0–15), while the value of f_2_ is greater than 50% (50–100) [25,30].

## 3. Results

### 3.1. Solubility Test

The maximum dose strength for NF is 200 mg (according to data on https://www.drugs.com/international/naftidrofuryl.html). Using the equilibrium solubility method, we showed a high solubility of NF in all buffers pH range 1.2–6.8. The lowest value of solubility (about 169 mg/mL) was observed on acetic acid conditions (pH 4.5), and the highest value (about 290 mg/mL) was obtained in pH 6.8. Thus, the calculated minimum dose/solubility ratio (D/S) was lower than 250 mL, and the dose number (D_0_) was less than 1 in each medium. The results of the equilibrium solubility are shown in Table 3.

### 3.2. Permeability Assay on Caco-2 Cell Line

To assess the permeability of NF across the Caco-2 monolayer, we selected CAF classified as the reference standard for highly permeable and BCS class I substances [24,25].

#### 3.2.1. Effect of NF and CAF on Caco-2 Cells Viability

**Figure 1:**
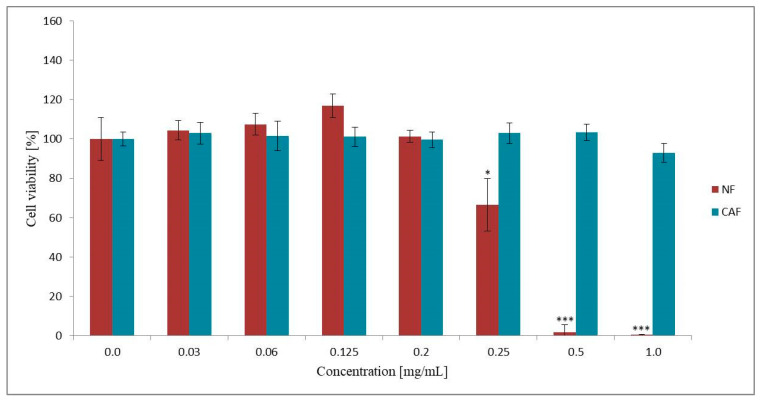
The effect of NF and CAF on Caco-2 cell viability. Results of the three independent replicates are presented as the mean ± SD. *** *p* < 0.001 and * *p* < 0.05.

The concentrations of the drugs to be used for the permeability tests were established by using the MTT viability test. The highest concentration of the test compounds with no cytotoxic effect on Caco-2 cells was preferred. To evaluate the cytotoxic activity of NF and CAF, the Caco-2 cells were exposed for two hours to the concentration ranges of each compound from 0.0 mg/mL to 1.0 mg/mL. The MTT results (Figure 1) showed that NF caused a more cytotoxic effect, as compared to CAF since only the highest concentration of CAF (1.00 mg/mL) slightly decreased the viability of Caco-2 cells (~92%). In contrast, 0.250 mg/mL of NF concentration caused down-regulation of cell viability to ~67%. Hence, in the permeability study, we used 0.200 mg/mL and 0.125 mg/mL of NF concentrations as they were not cytotoxic in the Caco-2 cell line.

#### 3.2.2. Caco-2 Permeability Assay

The intestinal epithelial Caco-2 cell culture model was applied to determine the permeability of NF and CAF across the intestinal barrier. TEER value measurements monitored the integrity of the Caco-2 monolayers during 22-day cultivation. The TEER parameter stabilized at 614 ± 24 Ω cm^2^ on the 7th day of culture. Only monolayers with TEER values beyond 600 Ω cm^2^ were used in the transport experiments. Bidirectional transport was analysed at two NF concentrations (0.125 mg/mL and 0.200 mg/mL) to determine the drug dose permeability relationship. CAF, as a reference intestinal permeable compound, was transported across the Caco-2 barrier at a concentration of 0.500 mg/mL. No cytotoxicity of NF and CAF at the concentrations tested after 2-h transport was observed. The TEER measurement indicated the high integrity of Caco-2 cell monolayers before and after permeability experiments (data not shown).

**Figure 2:**
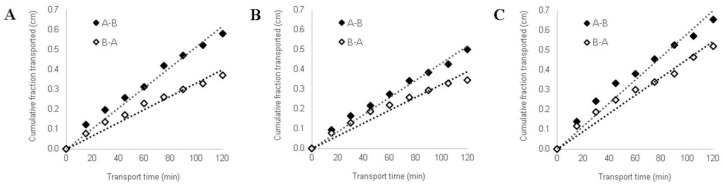
The experimentally (◊) and theoretically (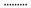) determined “cumulative fraction transported” of naftidrofuryl oxalate (NF) and caffeine (CAF) versus time in bidirectional A–B and B–A transport across Caco-2 cell monolayer. NF concentrations in donor compartments were established at 0.125 mg/mL (**A**) and 0.200 mg/mL (**B**). CAF transport was analyzed at an initial concentration of 0.5 mg/mL (**C**).

Based on HPLC data, the NF and CAF transport kinetics and the apparent permeability coefficient (P_app_) values were evaluated. The “weighted normalized cumulative amount of transported compound” for simplicity commonly named in the literature “cumulative fraction transported” (CFT) in the receiver compartment of the Caco-2 experimental model increased linearly with time, independently on transport direction (Figure 2). The time-course analysis of NF and CAF intestinal permeability showed that CFT was significantly higher in the absorptive direction (A-B transport) than in the secretory direction (B–A transport). This was also confirmed by the Papp values determined for NF and CAF transported in A–B and B–A direction (Table 4).

The results obtained from transport experiments showed that in vitro permeability of NF across the Caco-2 intestinal barrier is relatively high. The P_app_ values estimated for A–B transport of NF at both concentrations were higher than 100 × 10^−6^ cm/s. Permeability coefficients for B–A transport were calculated at 84.1 × 10^−6^ cm/s and 78.8 × 10^−6^ cm/s for NF at concentrations of 0.125 mg/mL and 0.200 mg/mL, respectively (Table 4). The results obtained from transport studies confirmed high intestinal permeability of CAF in both transport directions.

### 3.3. Dissolution Profiles Comparison

**Figure 3:**
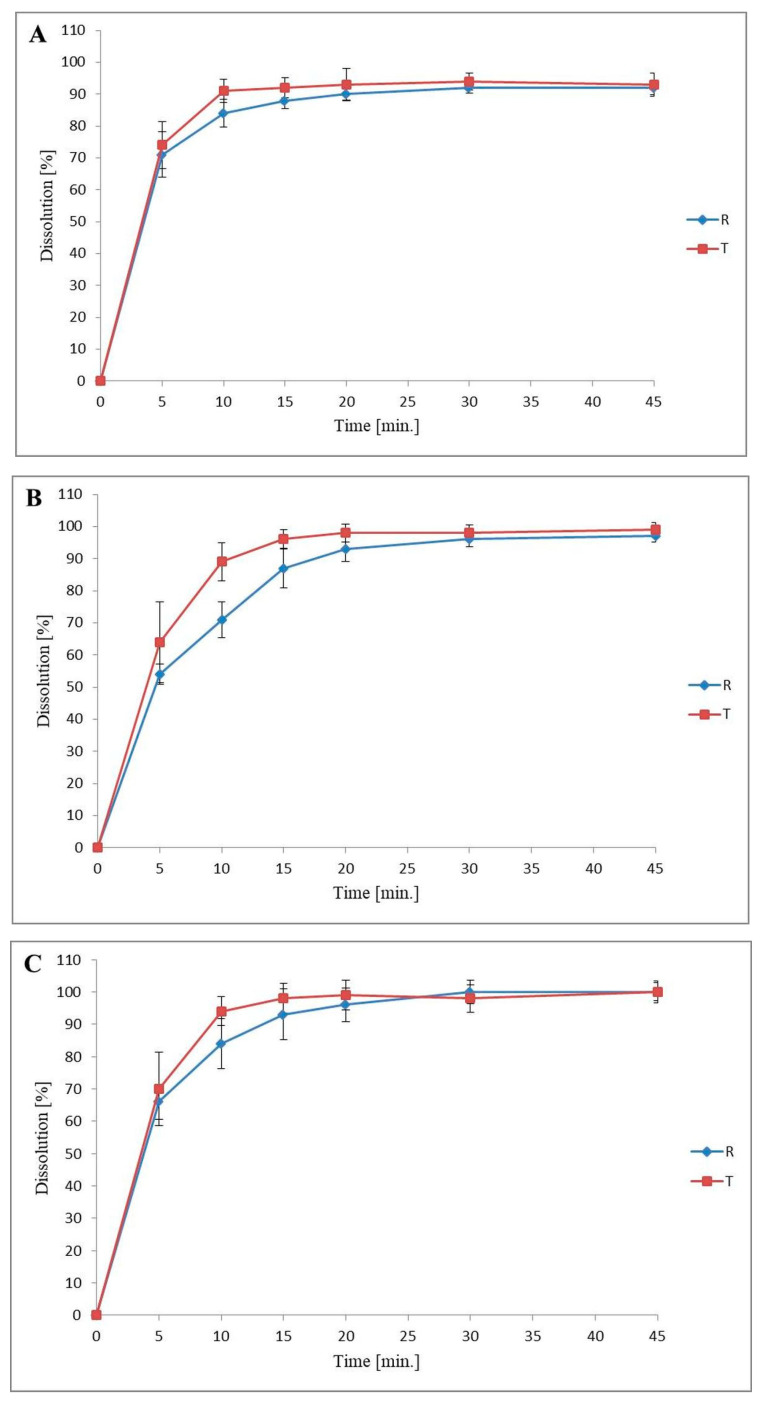
Comparative assessment of the in vitro release profiles of NF from in-house NF capsules (T, tested product) and Praxilene (R, reference product) in three different media 0.1 M HCl (**A**), acetate buffer (**B**), and phosphate buffer (**C**). Each time point on the curves shows the aggregated (*n* = 12) average percentage of NF release level from these products.

The in vitro dissolution testing was performed based on EMA guideline [24]. NF was released from both products, Praxilene, and the in-house NF capsules, very rapidly > 85% in less than 15 min. The dissolution rate of NF capsules was independent of pH and buffer species of the dissolution media. We observed a slightly faster release NF from in-house NF capsules than a reference product (Praxilene). The results of dissolution profiles of NF from in-house NF capsules and Praxilene are shown in Figure 3.

According to EMA, the confirmation of two curves (dissolution profiles) is not required for the IR product (≥85% in 15 min) [24]. Nevertheless, the mathematical estimation with standard statistical analysis was performed. The raw data (% RSD of the results in early dissolution time points, up to 10 min., of not more than 20%, and no more than of 10% in the subsequent time points) are consistent with the EMA guideline (Table 5) [24].

The statistical evaluation of the two profiles similarity was performed using the two factors (f_1_, f_2_) method. The summary of the results is presented in Table 6. Our results showed a mathematical similarity between the dissolution profiles of the test (in-house NF capsules) and the reference product (Praxilene) for each medium (f_2_ ≥ 50); the EMA guideline [24] was met. The release profiles showed the most significant similarity in pH 1.2 and 6.8 (f_1_ ≤ 15, f_2_ ≥ 50), while in pH 4.5, they were on the border of the accepted criteria (f_2_ = 51, f_1_ = 11).

## 4. Discussion

The BCS-based biowaiver guidelines take into account the rate of APIs release from the solid oral dosage forms, their solubility, and the in vitro transepithelial transport, thus describing the first stages of a drug LADME (liberation and absorption) [24,25]. To the best of the authors’ knowledge, there is no data concerning the classification of NF to the BCS. NF is known to dissolve well in aqueous media and ethanol [5,27]. Based on this information, NF might be suggested to belong to BCS class I (high solubility, high permeability) or BCS class III (high solubility, low permeability). Thus, the present study aimed to determine the aqueous solubility of NF and its permeability through Caco-2 monolayers.

The determination of NF solubility was performed using the equilibrium solubility method. Based on EMA guideline, a substance is considered well soluble when the highest therapeutic dose dissolves in 250 mL or less of aqueous media within the pH range of 1.2–6.8 at 37 ± 1 °C [24]. According to FDA guideline, the solubility test should be evaluated based on the ionization characteristics of the tested substance in pH = pKa, pH = pKa + 1, and pH = pKa−1 [25]. The pKa value of NF has been reported as 8.2 [6,19], which is above the human gastrointestinal tract (GIT) physiological range. Therefore, we did not extend the pH condition above the physiological range. In the solubility studies, we used three media in ranges of gastrointestinal pH (1.2, 4.5, and 6.8) and the highest 200 mg NF dose strength. We showed that the minimum solubility of the NF highest single dose was 169.0 mg/mL at pH 4.5. The calculated D/S ratio was 1.2 mL, thus far below the critical value of 250 mL. Our results indicate that NF is a highly soluble substance in the entire examined pH range. The highest solubility value at pH 6.8 suggests that NF might be well soluble in intestinal pH conditions positively affecting drug absorption from the GIT.

According to BCS-based protocols, the permeability of a drug substance throughout the GIT can be assessed by in vitro methods using a monolayer of cultured epithelial cells [24,25]. Hence, to determine the absorption of NF, the permeability test using the Caco-2 cell line was performed, and permeability coefficients (P_app_) were determined in both directions. In the present study, the permeability test of CAF was performed to confirm the suitability of the Caco-2 cell line system applied to determine transepithelial transport. CAF is recognized as a BCS reference substance [24,25] for which high permeability (P_app_ = ~50 × 10^−6^ cm/s; concentration 0.06 mg/mL) has been well documented [31,32]. Since we observed high transepithelial transport of CAF in both directions, the usefulness of the Caco-2 cell model was confirmed. The permeability study of CAF was performed only at the highest concentration (0.5 mg/mL) without cytotoxic effects against Caco-2 cells. According to BCS requirements for permeability analysis, the concentration of drug substance should be used in the amount of 0.01, 0.1, and 1 times its highest strength dissolved in 250 mL [24,25]. Assuming the NF 200 mg as the highest dose dissolute in 250 mL, we obtained a concentration range as follows: 0.8 mg/mL, 0.08 and 0.008 mg/mL. We examined the cytotoxicity of NF starting on the highest 1.0 mg/mL concentration. The 0.250 mg/mL of NF concentration was observed to cause down-regulation of cell viability to ~67%. Therefore, the bidirectional transport of NF was investigated at the two highest non-cytotoxic concentrations (0.125 mg/mL and 0.200 mg/mL), in the order of magnitude close to 0.08 mg/mL. We did not perform the permeability study of NF in the lowest concentration (0.008 mg/mL) since the analytical range of the NF assay method was validated for 0.01–0.3 mg/mL concentrations. According to literature data, when P_aap_ is lower than 1 × 10^−6^, the substance is considered as poorly permeable (0–20%). Concomitantly, the highly permeable drug is characterized by P_aap_ > 10 × 10^−6^ [29,33]. Our results showed that the permeability of NF across the cells barrier is quite high since the estimated values of P_app_ for A-B transport direction in both doses were larger than 100 × 10^−6^ cm/s. Furthermore, we observed that the P_app_ values of NF within its non-cytotoxic concentrations were almost as good as highly permeable CAF results. Hence, a rapid and total in vivo epithelial transport of NF might be suggested.

In general, we suggest that NF might be assigned to BCS I class due to its high solubility and high permeability. However, to fully comply with BCS requirements, the permeability tests should be validated using several additional reference substances that represent different levels of permeability (high, moderate, low, zero, and efflux substrates).

A drug product is eligible for biowaiver procedure when the substance belongs to BCS I or III class and is an immediate-release oral dosage form [24,25]. Thus, we also examined the in vitro dissolution rate of in-house prepared NF capsules in accordance with the Praxilene^®^ 100 mg formulation as a reference drug. The Praxilene product has a relatively simple composition with only two excipients (talc and magnesium stearate) that allowed us to closely define the quantity of each excipient in the dosage form. In our study, we showed that both products fulfil the IR requirements (>85% in not less than 15 min). Additionally, the similarity of the curves was confirmed statistically (f_1_ ≤ 15; f_2_ ≥ 50) in all media used in the range of pH 1.2–6.8. Thus, we conclude that multisourced excipients used for NF capsules preparation had no or minimal effect on its release rate. Our observations are in agreement with Flangan et al. since the excipients used for BCS I class drugs have been generally shown not to affect the intestinal absorption rate [34]. Moreover, the IR status of analyzed products indicates high pharmaceutical availability of NF throughout the GIT. Thus, we suggest that NF oral dosage form in comparison with Praxilene as a reference product meets the pharmacological equivalence criteria.

## 5. Conclusions

NF is a highly soluble substance within a wide pH range of physiological GIT conditions and highly permeable across Caco-2 cell monolayer. Thus, in the present study, we suggest that NF might be classified into BCS class I. Furthermore, we showed high dissolution rate of encapsulated NF based on Praxilene^®^ 100 mg composition and, therefore, it might be considered as an IR. To the best of the authors’ knowledge, this is the first attempt to classify NF based on BCS requirements. The experimental design of this study was based mainly on the BSC guidelines for the pharmaceutical industry. Hence, our paper could be useful for further developmental studies on NF containing drugs.

## Figures and Tables

**Table 2:** Composition of the developed in-house NF capsules.

Ingredients	Quantity (Per One Capsule)	Function
Naftidrofuryl oxalate	100 mg	active ingredient
Talc	rest of the unit filling weight	filler
Magnesium stearate	up to 0.5% of the unit filling weight	lubricant

**Table 3:** Solubility results of 200 mg NF in three different pH media at 37 °C. Results of the three replicates are presented as the mean ± SD.

Dose (D) (mg)	pH	Solubility (S) (mg/mL)	D/S Ratio (mL)	D_0_
200	1.2	279.00 ± 15.69	0.69	0.003
	4.5	169.00 ± 6.79	1.19	0.005
	6.8	290.40 ± 19.83	0.67	0.003

**Table 4:** Apparent permeability coefficients (P_app_) of naftidrofuryl oxalate (NF) and caffeine (CAF) determined in the Caco-2 intestinal barrier model (*n* = 3).

P_app_ × 10^−6^ (cm/s)			
Drug Substance	Concentration (mg/mL)	Apical-to-Basolaterial Transport	Basolaterial-to-Apical Transport
NF	0.125	131.9 ± 34.8	84.1 ± 4.7
NF	0.200	100.8 ± 15.1	78.8 ± 1.0
CAF	0.500	161.3 ± 19.7	126.5 ± 2.4

**Table 5:** The % RSD values for the in vitro release profiles of NF from in-house NF capsules (T, tested product) and Praxilene (R, reference product) in three different media 0.1 M HCl, acetate buffer, and phosphate buffer.

Time Point [min.]	0.1 M HCl pH 1.2 (%RSD)		Acetate Buffer pH 4.5 (%RSD)		Phosphate Buffer pH 6.8 (%RSD)	
	R	T	R	T	R	T
5	10.0	10.0	5.8	19.8	8.3	16.3
10	5.2	4.1	7.7	6.7	9.2	4.8
15	2.9	3.5	7.1	3.2	8.5	4.7
20	2.1	5.4	4.1	2.9	5.3	4.6
30	1.9	2.8	2.3	2.4	3.6	4.3
45	2.3	3.9	2.0	2.3	2.8	3.3

**Table 6:** Summary of f_1_ and f_2_ values for dissolution profiles curves in three different media.

Requirement	0.1 M HCl pH = 1.2	Acetate Buffer pH = 4.5	Phosphate Buffer pH = 6.8
Difference factor f_1_ (profiles are similar when f_1_ ≤ 15)	2	11	6
Similarity factor f_2_ (profiles are similar when f_2_ ≥ 50)	71	51	65
